# Comments about comments: peer review and the amazing editorial board of *Brain Communications*

**DOI:** 10.1093/braincomms/fcae029

**Published:** 2024-03-05

**Authors:** Tara L Spires-Jones

**Affiliations:** Edinburgh, UK

## Abstract

Our editor discusses our editorial board members, who come from eight countries on four continents, and wider issues of the peer review system.

## Graphical Abstract

**Graphical Abstract:**
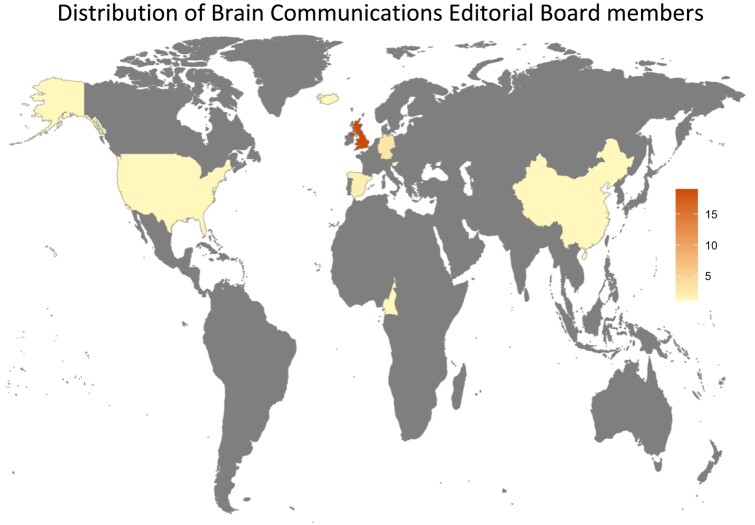

Welcome to Volume 6, issue 2 of *Brain Communications*. Fans of the journal may remember one of our early editorials outlining the work flow of papers submitted to us.^1^ Our associate editors play a key role in this process, donating their time and expertise to facilitate peer review of your submissions. Towards the end of 2023, submissions to our journal were rising to such an extent that we decided to invite more associate editors into our editorial board. In this editorial, I’d like to welcome the new members of the team and thank all editorial board members for their work on the journal. You can find the full list of our editors on our website (https://academic.oup.com/braincomms/pages/Editorial_Board). In addition to new members with expertise in areas popular with our authors (movement disorders, machine learning, mechanisms of neurodegeneration, genetics, molecular epidemiology, epilepsy, etc.), we are delighted to welcome associate editors based in many different parts of the world. Our board now has members from the UK, Germany, Spain, the USA, China, Cameroon, Slovenia, Iceland and France (see map in the associated Graphical Abstract).

The role of the academic editorial team is to invite peer reviewers with relevant expertise and make the decisions about whether to accept or reject papers. In our team, we work together to be sure these decisions are in line with our ethos to enhance rigour and reproducibility in translational neuroscience papers. Our scientific editorial team, composed of people with PhDs or MScs in neuroscience, checks each manuscript to be sure it complies with our policies on rigour, and as editor in chief, I check decisions to be sure we are not rejecting papers solely based on lack of novelty since we encourage replication studies.

Many in academia have been discussing a ‘peer review crisis’ because of difficulty journals like ours have in finding enough suitable experts willing to review each paper. And others are disgruntled at the system of peer review, which relies on academics doing the unpaid work of peer reviewing papers for journals that often make large profit margins.^2^ This frustration at the peer review system is prevalent on social media and in commentaries, but in surveys of scientists that I can find, the vast majority of people who responded think that peer review is an important part of the research ecosystem both for improving the work in papers and in feeling part of the research community.^3-5^ Undoubtedly, there are problems with the peer review system including the potential to introduce bias into publishing, which is hotly debated and poorly understood.^6^ The practice of scientists reviewing each other’s work dates back to the 18th century when the Royal Society started distributing reports to its members to ask them to veto publishing anything that could damage the reputation of the society. I may be an old-fashioned academic, but my view is that peer review still plays an important role in scientific communication. There is some evidence that peer review enhances the quality of reporting compared to preprints,^7^ and I think that peer review is an important part of community building and pushing our field forward.

At *Brain Communications*, we are trying to curate robust papers in the translational neuroscience space and facilitate constructive peer review. Several surveys and commentaries I’ve come across have indicated involving more early career researchers in the peer review process is important for the future of scientific publishing.^2,4,5^ We are doing our small part to facilitate this at *Brain Communications* through our reviewer academy. Everyone is welcome to watch and share the online training session run by our associate editor, Professor David Belin, who discussed with our reviewer academy members how to review papers constructively (https://www.youtube.com/watch?v=v0f_cc3m5ZM).

The cover image for this issue is courtesy of Danilo Negro and is associated with the scientific commentary by Negro and Opazo.^8^ It shows a 3D reconstruction of dendritic spines (red) from a live EGFP-labelled cortical neuron in a human brain slice culture imaged at 7 days *in vitro*. Segmentation and rendering were performed with 3dSpAn and ITK-SNAP. The human brain slice culture was generated by the Durrant lab and imaged in the Opazo lab using two-photon microscopy (University of Edinburgh).
